# Adjuvant Chemotherapy and Immunotherapy in Upper Tract Urothelial Carcinoma: Where Do We Stand?

**DOI:** 10.3390/cancers17193226

**Published:** 2025-10-03

**Authors:** Samuel Carbunaru, Jordan M. Rich, Katie S. Murray

**Affiliations:** Department of Urology, NYU Langone School of Medicine, New York, NY 10016, USA

**Keywords:** upper tract urothelial carcinoma, adjuvant chemotherapy, immunotherapy

## Abstract

**Simple Summary:**

In this review article, we provide an analysis of the current evidence supporting adjuvant chemotherapy and immunotherapy for upper tract urothelial carcinoma by discussing landmark trials including POUT, Checkmate 274, IMvigor 010, and AMBASSADOR. We also highlight future directions in the space by reviewing advances in cancer genetics and the emerging use of circulating tumor DNA.

**Abstract:**

Upper tract urothelial carcinoma (UTUC) is a rare malignancy that accounts for a small minority of all urothelial cancers. Historically, treatment recommendations for UTUC have been extrapolated from bladder cancer trials due to limited high-quality, UTUC-specific evidence. However, emerging data has shown how UTUC exhibits distinct biological, molecular, and clinical features compared to bladder cancer. In this piece, we provide an analysis of the current evidence supporting adjuvant chemotherapy and immunotherapy for UTUC. We discuss landmark trials such as the POUT trial for adjuvant chemotherapy, as well as pivotal trials such as CheckMate 274, IMvigor 010 and AMBASSADOR that examine the role of adjuvant immunotherapy for UTUC. Additionally, we briefly highlight advances in cancer genetics and the emerging use of circulating tumor DNA as a potential biomarker. While there has been significant progress made in adjuvant treatments for UTUC, substantial knowledge gaps remain. Clinical trials using UTUC-specific populations will be critical in improving outcomes and personalizing care for this patient population.

## 1. Introduction

Upper tract urothelial carcinoma (UTUC) is a rare urological cancer that accounts for approximately 5–10% of all urothelial carcinomas [1,2]. Due to the low incidence of UTUC, which limits recruitment for high-evidence randomized control trials (RCTs), much of the evidence behind adjuvant therapy for UTUC has been extrapolated from bladder cancer trials or as a subset of these trials [3,4]. Historically, UTUC has been considered the “twin tumor” of urothelial bladder cancer since they both arise from the transitional epithelium of the urinary tract. However, we now know that UTUC exhibits unique clinical, molecular, and prognostic characteristics [5,6]. For instance, in whole-gene sequencing studies, UTUC appears to have different frequencies in genetic mutations and unique molecular phenotypes compared to urothelial carcinoma of the bladder [7,8]. Additionally, UTUC is often detected at a more aggressive state, with about 60% of cases found to be locally advanced at diagnosis [9]. In this article, we perform an in-depth analysis of the evidence used to support recommendations for adjuvant chemotherapy and immunotherapy for UTUC, and discuss future directions in treatment therapies.

## 2. Adjuvant Chemotherapy

According to the most recent American Urological Association (AUA) guidelines, cisplatin-based neoadjuvant chemotherapy (NAC) is currently recommended for patients with high-risk UTUC undergoing nephroureterectomy or ureterectomy, particularly for those who are expected to have a reduction in renal function post-operatively that would disqualify them from adjuvant platinum chemotherapy [10]. There have been recent UTUC-specific trials that solidified the use of platinum-based chemotherapy in the neoadjuvant and adjuvant setting.

One of the early landmark studies that established NAC’s role in urothelial cancer was SWOG 8710, published in 2003 [11]. This phase III trial compared three cycles of MVAC (methotrexate, vinblastine, doxorubicin, and cisplatin) before radical cystectomy versus surgery alone, and found a significant improvement in median overall survival (OS) (77 months vs. 46 months). These findings have been recently tested in UTUC-specific populations in the ECOG A8141 trial [12] and NCT01261728 [13], which both found a ~60% downstaging rate and 14–19% complete pathological response rate after NAC.

Despite this growing body of evidence, NAC has not become routine in clinical practice and some reports estimate that only ~3% of high-risk UTUC receive NAC [14,15]. Many concerns surround how well patients will tolerate cisplatin-based NAC before surgery and whether it could accelerate renal decline. These issues have led some clinicians to favor adjuvant chemotherapy as the mainstay instead.

A major shift in the adjuvant treatment recommendations came from the POUT trial, a landmark phase III randomized controlled study that focused specifically on UTUC [16]. Published in 2020, POUT was the first trial to provide level 1 evidence for adjuvant chemotherapy in this disease. It enrolled 261 patients with non-metastatic, invasive or node-positive UTUC who had not received NAC. All underwent nephroureterectomy and were then randomized to either surveillance or four cycles of platinum-based adjuvant chemotherapy. Roughly 2 in 3 patients received cisplatin plus gemcitabine, but those with reduced kidney function (GFR <50 mL/min) were treated with carboplatin plus gemcitabine.

The trial’s primary endpoint was disease-free survival (DFS), with secondary endpoints including metastasis-free survival, overall survival (OS), toxicity, and quality of life. After a median follow-up of about 30 months, results strongly favored the adjuvant chemotherapy group. The hazard ratio (HR) for DFS was 0.45 (*p* = 0.0001), and the estimated 3-year event-free survival was 71% with adjuvant chemotherapy vs. 46% with surveillance.

Updated results published in 2024 showed that the DFS benefit held up at five years: 62% vs. 45% (HR = 0.55, *p* =001) [17]. There was also a possible signal for improved OS (HR = 0.6, *p* = 0.049) on univariate analysis, though that result was not statistically significant on multivariate analysis. The POUT trial changed the landscape for treatment in UTUC. Based on these findings, the AUA is now recommending offering adjuvant chemotherapy to eligible patients with locally advanced or node-positive UTUC after surgery who have not received NAC.

Some questions remain after the POUT trial. First, there is no direct evidence comparing the oncologic benefit of administering chemotherapy in the neoadjuvant vs. adjuvant setting. The authors initially considered designing the trial for neoadjuvant chemotherapy; yet after they discussed with patient focus groups and investigators, the authors opted to focus on adjuvant treatments as patients expressed strong preferences for potentially avoiding chemotherapy when given the choice and instead receiving it after surgical treatment. While the trial showed superiority of adjuvant chemotherapy compared to surgery alone, the question of whether chemotherapy is more beneficial in the neoadjuvant vs. adjuvant setting still remains unanswered. Second, while DFS is improved with adjuvant chemotherapy, and prior studies have shown a correlation between DFS and OS in bladder cancer populations, it is not entirely clear whether an improvement in DFS translates to a meaningful improvement for patients’ lives without clearly enhancing OS. Another issue is the role of carboplatin. In bladder cancer, perioperative carboplatin is not recommended, as no strong trial data currently supports its use. Despite this, in POUT, over one-third of patients received carboplatin instead of cisplatin due to poor renal function. The authors of the study noted that DFS outcomes did not significantly differ between those who received cisplatin versus carboplatin. However, the trial was not designed or powered to detect that kind of difference. It is possible that important distinctions between the two regimens were missed. Future research should look more closely at this, particularly since many real-world UTUC patients cannot tolerate cisplatin, especially after nephroureterectomy. Understanding the true comparative effectiveness of these agents could help refine treatment recommendations even further.

## 3. Adjuvant Immunotherapy

The role of adjuvant systemic immunotherapy in UTUC has been evolving in the last decade. Most of the evidence comes from two large RCTs: CheckMate 274 and IMvigor 010, which mostly recruited bladder cancer patients, yet their findings were extrapolated to UTUC populations.

CheckMate 274 trial was a phase III RCT that randomized 709 patients who underwent radical extirpative surgery for urothelial cancer to receive either adjuvant nivolumab or placebo for up to one year post-surgery [18] with a primary endpoint of DFS. It is worth mentioning that 53% of patients in the nivolumab group and 56% in the placebo group discontinued the trial regimen (most commonly due to disease recurrence). About ~43% of patients had received neoadjuvant cisplatin-based chemotherapy standard of care. While most participants’ primary tumor was located in the bladder, 21% of patients presented with primary UTUC (*n* = 149, 53 with ureteral tumors and 96 with renal pelvis tumors). The trial showed a 10-month increase in median DFS in the nivolumab group (20.8 months vs. 10.8 months). At 12 months, 62% of patients in the nivolumab group were disease-free (compared to 46% in the placebo group, *p* < 0.001). This difference was even more pronounced in patients with a PD-L1 expression >1%, where 67% of patients were disease-free at 12 months. Subgroup analyses were performed for non-bladder primary tumors. The HR for disease recurrence or death was not significant for either renal pelvis tumors (HR 1.23, 95% confidence interval (CI): 0.67–2.23) or ureteral tumors (HR 1.56, CI: 0.70–3.48). However, given the small sample size of each subgroup study arm (40–50 patients in each study arm for renal pelvis tumors, and 20–30 patients for ureteral tumors), the study was not powered to detect any differences in these subgroups. The authors did not combine both groups to perform an “UTUC population” analysis. In terms of tolerability, grade 3 or higher treatment-related adverse events were reported in 17% of patients in the nivolumab group compared to 7% in the placebo group. About 12% of patients discontinued the nivolumab regimen due to adverse events.

The IMvigor 010 trial was another large phase III RCT where 809 patients with high-risk urothelial carcinoma were randomized to either adjuvant atezolizumab or observation [19]. They included patients who received NAC and had pathological stage T2-4a/pN+ tumors after surgery, or those who did not receive NAC and had T3-4a/pN+ tumors. This trial recruited a much smaller cohort of UTUC, which comprised ~7% (*n* = 54) of the study’s population. The authors did not differentiate between ureteral and renal pelvis tumors in the UTUC population. The trial did not meet its primary endpoint, as the median DFS was 19.4 months in the atezolizumab group compared to 16.6 months in the observation group (HR 0.89, CI: 0.74–1.08, *p* = 0.24). Subgroup analysis looking at patients with upper tract tumors showed a non-statistically significant DFS HR between treatment groups of 1.25 (CI 0.57–2.74). However, it is important to note the small cohort size of this UTUC subgroup (25–29 patients in each treatment group), which likely limited the power to identify any noticeable difference. About a third of patients experienced an adverse event that led to a dose interruption, and 16% of patients had a grade 3 or higher treatment-related adverse event, with pyrexia (2%) being the most common.

Another more recent phase III cooperative group United States-based trial (AMBASSADOR) randomized patients to adjuvant pembrolizumab after extirpative surgery for UCC [20]. The trial was closed prematurely after the standard of care recommendation changed in favor of adjuvant nivolumab based on CheckMate 274 data that was published. Despite this, enrollment was at 96% at the time of study closure. The study population included 22% of enrolled patients with UTUC (*n* = 154, 93 with renal pelvis tumors and 61 with ureteral tumors). In the entire cohort, the median DFS more than doubled in the pembrolizumab group (29 vs. 14 months); however, over half the patients in the pembrolizumab arm experienced a grade ≥ 3 adverse event, with 24.8% of patients having a treatment-related adverse event. There was not a statistically significant difference between each treatment group on subgroup analyses for UTUC patients (including when looking at the total UTUC population, and when stratifying patients into renal pelvis and ureteral tumors. Interestingly, there was a tendency for patients with renal pelvis tumors to have higher rates of disease progression or death in the pembrolizumab group (HR 1.96, CI 0.92–4.17), which was not statistically significant. There were only 11 patients in the renal pelvis tumors observation group, so the study was likely underpowered to perform this analysis. There are additional ongoing trials looking into other immunotherapy agents in UTUC, such as the EA8192 and also iNDUCT trials, both assessing combination NAC with neoadjuvant durvalumab in UTUC-specific populations [21].

A summary of these three trials can be seen in Table 1. Given the conflicting results of these trials and low recruitment of UTUC patients that constrain the generalizability of their findings, the current AUA guidelines recommend adjuvant platinum-chemotherapy over adjuvant immunotherapy for eligible patients who did not receive neoadjuvant chemotherapy (NAC). However, there seems to be a role for adjuvant nivolumab in patients unable to tolerate platinum-based chemotherapy or those with high-risk pathology even after NAC.

## 4. Future Directions

As we improve our understanding of UTUC, novel findings in cancer genomics are shaping our ability to potentially offer individualized treatment strategies in the future. We continue to make advances in our understanding of the unique genetic profiles of UTUC and how it differs from bladder urothelial carcinoma on a molecular level. For instance, certain mutations in MYC, KMT2D, and FGFR3 are more frequently found in UTUC, while TP53, RB1, TERT, and ERBB2 mutations are more commonly seen in bladder cancer [22,23]. Another key distinction is the strong association between UTUC and Lynch syndrome, an inherited cancer syndrome caused by germline mutations in DNA mismatch repair (MMR) genes [22]. UTUC ranks as the third most common cancer in people with Lynch syndrome. These patients tend to have much higher rates of microsatellite instability and MMR deficiency than those with bladder cancer [24], which poses important implications for therapeutics. Tumors with microsatellite instability or MMR deficiency often have a higher number of neoantigens, making them more immunogenic and more likely to respond to immune checkpoint inhibitors like PD-1 or PD-L1 blockers [25]. Paradoxically, UTUC typically shows lower levels of PD-L1 expression and tumor mutational burden than bladder cancer, both of which usually predict weaker responses to immunotherapy [22]. However, in the context of Lynch syndrome, the presence of microsatellite instability and MMR deficiency may outweigh those predictive markers and still result in meaningful responses to immune checkpoint inhibition [26]. Thus, UTUC patients with Lynch syndrome are likely far better candidates for immune checkpoint inhibition than those without Lynch syndrome. These differences highlight the need for a more individualized approach to the management of UTUC. Specific focus on genetics, molecular biomarkers and immunologic profiling will be crucial in shaping treatment paradigms.

The clinical application of our enhanced understanding of cancer genomics can be seen with the development of targeted adjuvant therapies based on genomic expression profiles. One example of this is antibody–drug conjugates, such as disitamab, an anti-HER2 antibody. HER-2 is a subclass of tyrosine kinase receptors that has been associated with tumor growth in multiple cancers, including UTUC [27]. Distitamab has been previously studied in combination with pembrolizumab in patients with metastatic UTUC [28]. Its application as an adjuvant treatment for UTUC is still in the early phases, but results from phase II trials seem promising. Liu et al. recently published their preliminary findings from a single-arm prospective trial on patients receiving disitamab combined with toripalimab (anti-PD-1 monoclonal antibody) after radical surgery with pT2-4NanyM or those with stage N1-2M0 that did not receive neoadjuvant therapy [29]. There were 45 patients recruited, out of which 95% were stage T2 and all had HER-2 overexpression. The 1-year DFS was 90% (median DFS has not been reached). Enhanced insight into each patient’s genomic expression will likely play a pivotal role in providing personalized therapies.

Circulating tumor DNA (ctDNA) has gained popularity in the last decade as a biomarker that can not only aid in disease screening and recurrence monitoring, but also potentially predict treatment response [30]. In muscle-invasive bladder cancer, several studies have shown how both baseline ctDNA and ctDNA clearance are associated with oncological outcomes after neoadjuvant and adjuvant therapies [31,32,33]. On a recent exploratory analysis of the KEYNOTE-361 trial, Powles et al. performed an in-depth analysis of ctDNA dynamics after chemotherapy or immunotherapy, and its association with oncological outcomes in advanced urothelial carcinoma [34]. The cohort was mostly composed of bladder cancer patients, but ~20% of patients had UTUC. Over 80% of patients were ctDNA-positive; however, this number varied depending on the approach used for ctDNA detection. After treatment, chemotherapy patients appeared to have a more pronounced impact on ctDNA levels, with 41% of patients demonstrating ctDNA clearance, compared to 11% of patients in the immunotherapy group. However, baseline ctDNA levels and post-treatment ctDNA reduction appeared to have a stronger predictive validity in the immunotherapy group when assessing radiological response and clinical metrics, particularly overall survival. The authors then further stratified patients by tissue tumor mutational burden (tTMB) and PD-L1 expression, and found that those with high tTMB and high PD-L had the most dramatic response in ctDNA reduction in the immunotherapy group. This comprehensive study shed light on the potential implications of using ctDNA as a prognostic predictor of treatment response with either systemic therapy. There are still many unanswered questions and challenges that will need to be addressed such as discrepancies in ctDNA testing platforms and understanding how ctDNA kinetics varies by treatment; yet, ctDNA appears to be a promising biomarker that might enable more personalized treatment decisions for UTUC in the future.

## 5. Conclusions

There have been significant advances in our understanding of UTUC over the last decade. We historically assumed that all urothelial carcinomas have similar features; however, we now know that UTUC demonstrates unique molecular and clinical characteristics, making it deserving of its own trials and exploration. It is important to be aware of the evidence behind recommendations of adjuvant therapy for UTUC. While there have been UTUC-specific trials for adjuvant platinum-based chemotherapy, the evidence behind adjuvant immunotherapy has been extrapolated from trials that mostly included bladder cancer patients. As we continue to make progress in our understanding of this disease, future UTUC-specific trials will be helpful to optimize adjuvant treatment strategies and improve patient outcomes.

## Figures and Tables

**Table 1:** Landmark Trials of Adjuvant Immunotherapy in UTUC.

Study (Year)	No. Patients	Percentage of UTUC Patients	Inclusion Criteria	Cohorts	Median DFS	Grade 3+ Treatment- Related Adverse Events
**CheckMate 274** (2021) [18]	709	149 (21.0%)	T2-4a/pN+ after NAC or T3-4a/pN+ AND no NAC	Nivolumab vs. Placebo q2 weeks for up to 1 year	Nivolumab: 20.8 months Placebo: 10.8 months (*p* < 0.001)	63 (17.9%)
**IMvigor 010** (2021) [19]	809	54 (6.7%)	T2-4a/pN+ after NAC or T3-4a/pN+ AND no NAC	Atezolizumab vs. observation q3 weeks for up to 1 year or 16 cycles	Atezolizumab: 19.4 months Observation: 16.6 months (*p* = 0.24)	63 (16.2%)
**AMBASSADOR** (2025) [20]	702	154 (21.9%)	≥T2/pN+ and/or microscopic positive surgical margins after NAC ≥T3/pN+ or microscopic positive surgical margins AND no NAC	Pembrolizumab vs. observation q3 weeks for up to 1 year	Pembrolizumab: 29.6 months Observation: 14.2 months (*p* = 0.003)	82 (24.8%)
